# The effect of litter size relative to functional teat count on lactating sow and litter performance

**DOI:** 10.1093/tas/txaf161

**Published:** 2025-12-05

**Authors:** Abigail K Jenkins, Sierra M Collier, Joel M DeRouchey, Mike D Tokach, Jason C Woodworth, Katelyn N Gaffield, Jordan T Gebhardt, Robert D Goodband, Kyle F Coble, Paul J Corns, Jimmy Karl, Tag Bradley

**Affiliations:** Department of Animal Sciences and Industry, College of Agriculture, Kansas State University, Manhattan, KS, 66506-0201, United States; Department of Animal Sciences and Industry, College of Agriculture, Kansas State University, Manhattan, KS, 66506-0201, United States; Department of Animal Sciences and Industry, College of Agriculture, Kansas State University, Manhattan, KS, 66506-0201, United States; Department of Animal Sciences and Industry, College of Agriculture, Kansas State University, Manhattan, KS, 66506-0201, United States; Department of Animal Sciences and Industry, College of Agriculture, Kansas State University, Manhattan, KS, 66506-0201, United States; Department of Animal Sciences and Industry, College of Agriculture, Kansas State University, Manhattan, KS, 66506-0201, United States; Department of Diagnostic Medicine/Pathobiology, College of Veterinary Medicine, Kansas State University, Manhattan, KS, 66506-0201, United States; Department of Animal Sciences and Industry, College of Agriculture, Kansas State University, Manhattan, KS, 66506-0201, United States; JBS Live Pork, Greeley, CO, 80634-9039, United States; JBS Live Pork, Greeley, CO, 80634-9039, United States; JBS Live Pork, Greeley, CO, 80634-9039, United States; JBS Live Pork, Greeley, CO, 80634-9039, United States

**Keywords:** cross-fostering, functional teat count, lactating sow, litter size, pre-weaning performance

## Abstract

A total of 1005 sows and their litters were used to evaluate the effect of initial litter size relative to functional teat count on sow and litter performance. Sows were blocked by parity (1, 2 to 4, or 5+) and functional teat count (≤13, 14 to 15, or ≥16) categories and allotted to one of four treatments with 251 or 252 replications. Treatments consisted of 1 less pig than functional teats (−1), same number of pigs as functional teats (0), 1 more pig than functional teats (+1), or 2 more pigs than functional teats (+2). Pigs were individually weighed after cross-fostering and before weaning. Pigs born <0.9 kg were fostered onto sows not included in this study. Replacement pigs were not added to litters after a removal or mortality. Sow body weight (BW), caliper score, and backfat depth were collected at farrowing house entry and weaning (d 22). Parity category, treatment, and their interaction were fixed effects in the model along with teat category. As litter size relative to functional teat count increased, sows lost more BW and caliper units, but culling rate due to non-conception decreased (linear, *P ≤* 0.038). Litter size and weight increased (linear, *P <* 0.001) as initial litter size relative to functional teat count increased at d 2 and weaning. Litter average daily gain (ADG) exhibited a quadratic relationship (*P = *0.045) where −1 and +2 sows had numerically greater litter ADG compared to 0 and +1 sows. Mean pig weaning BW (linear, *P <* 0.001) and pig ADG decreased (quadratic, *P = *0.042) and removals and mortality (d 2 to weaning) increased (linear, *P <* 0.001) as initial litter size relative to functional teat count increased. Pigs weaned per sow per year (PSY) increased (linear, *P <* 0.001) as initial litter size relative to functional teat count increased. Wean-to-estrus interval (WEI) exhibited a quadratic relationship (*P = *0.049) where 0 sows had longer WEI compared to +2 sows with −1 and +1 sows intermediate. Subsequent farrowing rate did not differ; however, subsequent liveborn increased (linear, *P = *0.017) and total born tended to increase (linear, *P = *0.061) as previous litter size relative to functional teat count increased. In conclusion, sows with 1 less pig than functional teats after cross-fostering had the lowest piglet mortality and sow BW loss and greatest piglet weaning BW. However, sows with 2 more pigs than functional teats after cross-fostering had the greatest number of pigs weaned per litter, litter WW, and PSY.

## Teaser

Increasing initial litter size relative to functional teat count increased litter weaning weight, pigs weaned per litter, and pigs weaned per sow per year; however, it also decreased individual pig weaning weight and increased pre-weaning mortality and removals.

## Introduction

The modern hyperprolific sow often gives birth to more pigs than the number of functional teats that she possesses. Recent benchmarking reports for United States swine production systems have reported that the top 10% of farms are achieving an average liveborn per litter of 15.7 pigs (PigChamp 2024). However, recent research has shown that sows in some commercial swine herds have average functional teat counts of 13.9 (Obermier et al.2023). This imbalance in liveborn pigs and functional teat count requires alternative rearing strategies such as artificial rearing, fostering extra pigs onto nurse sows, or allowing sows to nurse more pigs than they have functional teats. Artificial-rearing systems involve housing piglets in specialized enclosures that provide warmth, milk replacer, and solid feed, allowing them to be removed from the sow between 2 and 14 d of age (Baxter et al.2013). While these systems can help manage surplus piglets, they have been associated with increased piglet tail lesion scores (Schmitt et al.2019), impaired immune function (Han et al.2024), and inconsistent effects on growth performance (Vergauwen et al.2017). Alternatively, a nurse sow is a sow whose own litter has been weaned or removed and is then replaced with a new litter of pigs (Alexopoulos et al.2018). This practice can reduce overall farm productivity due to lower milk yield (Kobek-Kjeldager et al.2020), greater culling rates (Osotsi et al.2024), and increased biosecurity risks by disrupting the all-in/all-out system and facilitating disease spread (Garrido-Mantilla et al.2020). In addition, nurse sow reduce the number of sows that can farrow because they require keeping empty farrowing stalls available for their transfer (Osotsi et al.2024).

Given the limitations and challenges associated with artificial rearing or using nurse sows, some producers opt to allow sows to nurse more piglets than they have functional teats. However, existing literature on this practice is equivocal and thus, the optimal pig-to-functional-teat ratio to enhance litter performance and reduce pre-weaning mortality (PWM) and effects on subsequent reproduction remains unclear. Zanin et al.(2024) observed no differences in PWM when litter size relative to teat count was increased from the same number of pigs as functional teat count to 1 more pig than functional teat count. In contrast, Vande Pol et al.(2021) observed that mortality increased from 7.7% to 17.9% when litter size was increased from 2 less pigs than functional teat count to 2 more pigs than functional teat count. To date, there are no studies using a large sample size that have examined a broad range of incremental increases in pig-to-functional-teat count ratios, while simultaneously assessing subsequent reproductive outcomes. Therefore, the objective of this study was to determine the effects of initial litter size relative to functional teat count on sow BW and body condition, litter performance, and subsequent reproductive performance under commercial conditions. We hypothesized that increasing initial litter size relative to functional teat count would increase litter size and weight at weaning but decrease piglet weaning weight (WW) and adversely affect sow body condition and subsequent reproduction.

## Materials and methods

### General

The protocol for this experiment was approved by the Kansas State University Institutional Animal Care and Use Committee (IACUC # 4915.01). This study was conducted at a commercial sow farm in northwest Texas. Sows were individually housed in an environmentally controlled and mechanically ventilated barn. Farrowing stalls measured 1.52 × 2.06 m. Each stall was equipped with a dry *ad-libitum* sow feed dispenser (SowMax, HogSlat, Newton Grove, NC) and an adjacent nipple waterer placed at sow shoulder height. An additional nipple waterer was located at the base of the stall, along with a heat lamp for piglets. Sows were provided *ad-libitum* access to a common sorghum-soybean meal-based lactation diet formulated to 1.05% standardized ileal digestible Lys throughout the duration of the study. Piglet needle teeth were not clipped during this study. Creep feed and supplemental milk were not offered to litters throughout this study.

### Animals and treatment structure

A total of 1005 sows (average parity 3.5, Line 1050, PIC, Hendersonville, TN) and their litters were used from June to August 2024. On approximately d 112 of gestation, sows were moved from the gestation facility into farrowing rooms which contained 148 farrowing stalls. Sows were blocked by parity category (1, 2 to 4, and 5+) and functional teat count category (≤13, 14 to 15, and ≥16 teats). Within block, sows were randomly assigned to treatment after farrowing was complete to equalize parity and functional teat count. Treatments were also balanced based on sow entry body weight (BW) and backfat depth. Treatments were based on initial litter size relative to functional teat count with treatment establishment (piglet movement) performed within 24 h of the completion of farrowing. Treatments consisted of: one less pig than functional teat count (−1), the same number of pigs as functional teat count (0), one more pig than functional teat count (+1), or two more pigs than functional teat count (+2). The determination and counting of functional teats and cross-fostering were performed by the same individual for all litters. In the context of this study, a functional teat was defined as one capable of producing sufficient milk to sustain a piglet (Alexopoulos et al.2018), is elongated and pointed with no visual defects, and can be suckled by the pig (Arend et al.2023). Therefore, any blunt teats, blind teats, or teats connected to mammary glands with severe edema were considered non-functional. Additionally, functional teat count was determined after farrowing occurred which gives more certainty in the number of functional teats when compared to counting at the time of entry into the farrowing house (Alexopoulos et al.2018). The average functional teat count in this study was 14.7. Pigs that were born less than 0.9 kg were not included in this study and were fostered onto sows where litters were provided specialized care per the standard operating procedure of this farm. To attain the correct number of pigs relative to functional teat count, average birthweight pigs were utilized for cross-fostering to maintain the normal bodyweight distribution within the litter.

### Measurements

A walk-on platform scale was used to collect sow BW at entry into the farrowing house and at weaning. Body weight loss after farrowing was modeled utilizing equations developed by Estrada et al.(2024). First, pre-farrowing adjusted body weight was calculated to account for conceptus growth between the time of weighing and farrowing. This was done using the equation: pre-farrow adjusted weight (kg) = (total pigs born × 0.039 × days from weighing to farrowing) + pre-farrow weight (kg). Next, conceptus weight was estimated as: conceptus weight (kg) = 0.137 + [1.329 × total pigs born × average pig birth weight (kg)]. Finally, sow body weight after farrowing was calculated by subtracting conceptus weight from the pre-farrow adjusted weight.

Sow backfat depth was measured at entry into the farrowing house and weaning at the last rib, 8 cm from the midline (Renco Lean Meter, S.E.C. Repro Inc., Golden Valley, MN). Sow caliper score (Knauer and Baitinger 2015) was measured at entry into the farrowing house and weaning at the last rib. Pigs were individually weighed after cross-fostering and the day before weaning. The average weaning age was 22 d. Beginning on d 3 of lactation, pigs that appeared gaunt, were not competitive at the udder, and were losing weight were deemed fall-behind pigs and removed from the litter. These pigs were only removed if two independent individuals of the research team agreed upon their removal. At the time of removal from the farrowing stall, all removals and mortalities were weighed and recorded. A replacement pig was not added to the litter in the incidence of a removal or mortality.

At weaning, pigs were classified into the following pre-determined BW categories: <3.6 kg, 3.6 to 4.5 kg, 4.5 to 5.4 kg, 5.4 to 6.4 kg, 6.4 to 7.3 kg, and >7.3 kg. The proportion of the litter that fell into each BW category was determined. In addition, the litter weight coefficient of variation (CV) was determined on d 2 and at weaning by dividing the standard deviation of pig weights within the litter by the average weight of pigs within the litter.

After weaning the insemination date was recorded using the CloudFarms System (BASF, Florham Park, NJ) and used to calculate wean-to-estrus interval (WEI) and percentage of sows bred by d 7 post-weaning. Sows culled for any reason or re-bred due to failure to conceive were not included in the subsequent farrowing data analysis. Subsequent farrowing data was obtained from the CloudFarms system. Estimated pigs weaned/sow/year (PSY) was calculated assuming a litters/sow/year of 2.43 which was the average of the farm at the time this study was conducted.

### Statistical analysis

Data were analyzed using the lme4 package of R (Version 4.0.0, R Foundation for Statistical Computing, Vienna, Austria) as a generalized randomized complete block design. Blocking structure accounted for parity category and functional teat count category. Parity categories were: first parity (*n* = 206), parity 2 to 4 (*n* = 487), and parity 5 and greater (*n* = 312). Functional teat count categories were: 13 or less (*n* = 163), 14 to 15 (*n* = 549), and 16 or more (*n* = 293). Sow (litter) served as the experimental unit. Treatment, parity category, and their interaction were included as fixed effects. Functional teat count category was also included in the model as a fixed effect. Preplanned linear and quadratic contrast statements were used to evaluate increasing initial litter size relative to functional teat count. Backfat depth at entry was included in the model as a covariate in the analysis of backfat depth at weaning and backfat change over lactation. The glm function was used to analyze count data using a negative binomial distribution with a log link function. The glm function (binomial distribution) using a logit link function was used to analyze proportion data, including pre-weaning mortality and removals. Percentage of sows bred by d 7 post weaning, percentage culled, and subsequent farrowing rate were analyzed using the glm function (binary distribution). Differences were considered significant at *P ≤* 0.05 and marginally significant at 0.05 < *P ≤* 0.10. All treatment × parity interactions were *P >* 0.05 unless otherwise indicated.

## Results

### Interactive effects of initial litter size relative to functional teat count and parity category

Backfat depth at entry was the only treatment × parity interaction (*P = *0.022) observed. Parity 1 sow backfat depth was numerically greater in +2 sows, whereas in parity 2 to 4 sows, backfat depth at entry was numerically greater in −1 sows, and numerically greater in +1 sows in parity 5+ sows (Table 1). There were no other interactions between initial litter size and sow parity category.

**Table 1.** Effect of initial litter size relative to functional teat count on sow lactation performance.^a^

						*P* =		
	Initial litter size +/− functional teat count^b^					Treatment		
Item	−1	0	+1	+2	SEM	Linear	Quadratic	Parity
**Sows, n**	251	252	251	251	…	…	…	…
**Parity**	3.5	3.5	3.5	3.5	0.14	0.968	0.906	…
**Sow BW, kg**								
** Entry**	275.2	274.4	275.3	274.7	1.96	0.921	0.943	<0.001
** After-farrow^c^**	245.0	244.9	245.5	245.2	1.94	0.868	0.966	<0.001
** Weaning**	238.2	238.6	235.4	234.8	1.78	0.080	0.749	<0.001
** On-test change (after-farrow to weaning)**	−6.8	−6.3	−10.0	−10.4	1.23	0.005	0.694	<0.001
** Lactation change (entry to weaning)**	−36.5	−35.8	−39.8	−40.1	1.24	0.006	0.686	<0.001
**Sow backfat depth, mm**								
** Entry^d^**	15.0	15.0	15.1	15.1	0.24	0.695	0.864	0.022
** Weaning^e^**	12.1	12.2	12.1	11.8	0.14	0.046	0.166	<0.001
** Lactation change (entry to weaning)^e^**	−3.0	−2.9	−3.1	−3.4	0.14	0.046	0.166	<0.001
**Sow caliper score**								
** Entry**	15.3	15.5	15.4	15.5	0.14	0.365	0.950	0.026
** Weaning**	13.1	13.2	12.9	12.7	0.16	0.055	0.254	<0.001
** Lactation change (entry to weaning)**	−2.3	−2.3	−2.5	−2.8	0.14	0.001	0.197	<0.001

a A total of 1005 mixed-parity sows (Line 1050, PIC, Hendersonville TN) and litters were used from the time of cross-fostering (approximately 24-h after farrowing) until weaning. Treatment and parity category and their interaction were used as fixed effects in the statistical model. Teat count category was included as a fixed effect in the model.

b Sows were allotted to treatment at approximately 24-h after farrowing. Sows were blocked by functional teat count and parity categories and assigned to 1 of 4 treatments: one less pig than functional teat count (−1), the same number of pigs as functional teat count (0), one more pig than functional teat count (+1), or two more pigs than functional teat count (+2).

c Body weight loss from entry to after farrow was modeled utilizing the equations developed by Estrada et al.(2024): Sow body weight after farrowing (kg) = pre-farrow adjusted weight (kg) - conceptus weight (kg). Pre-farrow adjusted weight (kg) = total pigs born  ×  0.039 × days until farrowing from time of weighing + pre-farrow weight (kg).

Conceptus weight (kg)  =  0.137 + 1.329 × total pigs born  ×  average pig birth weight (kg).

d Treatment × parity, *P = *0.022.

e Entry backfat depth included in statistical model as a covariate.

### Effect of initial litter size relative to functional teat count

Sow BW did not differ based on initial litter size relative to functional teat count at entry or after-farrowing. However, there was a tendency (linear, *P = *0.080) for decreased sow BW at weaning as initial litter size relative to functional teat count increased. Sow BW change (loss) during lactation increased (linear, *P ≤* 0.006) as initial litter size relative to functional teat count increased. Sow backfat depth at weaning decreased (linear, *P = *0.046) as initial litter size relative to functional teat count increased. Sow caliper score was decreased at weaning (linear, *P = *0.055) reflecting more caliper unit loss over lactation (linear, *P = *0.001) as initial litter size relative to functional teat count increased, similar to the change in backfat depth.

**Fig. 1.**
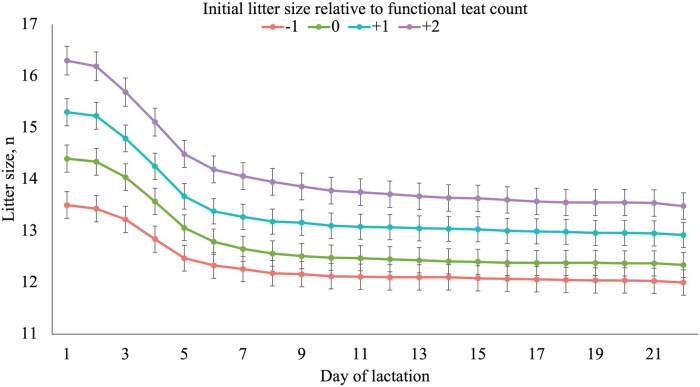
**Litter size by day of lactation within treatment**. Within each initial litter size relative to functional teat count treatment, the average litter size on each day of lactation is shown. A total of 1005 mixed-parity sows (line 1050, PIC, hendersonville TN) and litters were used from the time of cross-fostering (approximately 24-h after farrowing) until weaning. Sows were blocked by functional teat count and parity categories and assigned to 1 of 4 treatments: one less pig than functional teat count (−1), the same number of pigs as functional teat count (0), one more pig than functional teat count (+1), or two more pigs than functional teat count (+2). Data are expressed as LSM ± SE.

**Fig. 2.**
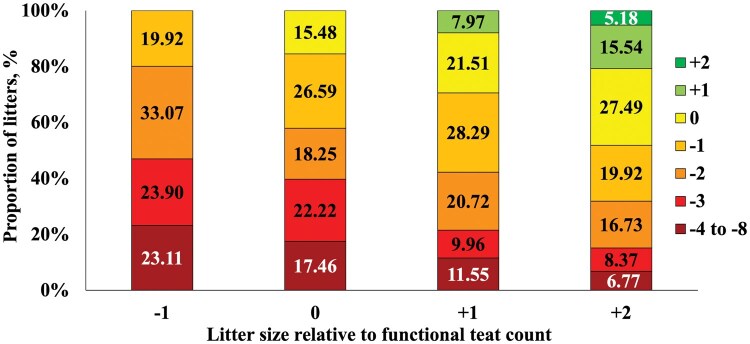
**Proportion of litters weaned relative to teat count**. Within each initial litter size relative to functional teat count treatment, the proportion of litters that were weaned at different pig counts relative to functional teat counts is shown. A total of 1005 mixed-parity sows (line 1050, PIC, hendersonville TN) and litters were used from the time of cross-fostering (approximately 24-h after farrowing) until weaning. Sows were blocked by functional teat count and parity categories and assigned to 1 of 4 treatments: one less pig than functional teat count (−1), the same number of pigs as functional teat count (0), one more pig than functional teat count (+1), or two more pigs than functional teat count (+2).

Litter size on d 2 and weaning increased (linear, *P <* 0.001) as litter size relative to functional teat count increased (Table 2). The reduction of litter size due to removals and mortality based on day of lactation followed a consistent trend, regardless of the initial litter size relative to the number of functional teats (Fig. 1). The number of pigs weaned per functional teat increased (linear, *P <* 0.001) as initial litter size relative to functional teat count increased. Notably, 47% of the −1 sows weaned three or more pigs below their teat count (Fig. 2). However, as initial litter size increased relative to teat count, the proportion of sows that weaned three or more pigs below teat count decreased, dropping to just 15% of +2 sows. In contrast, 48% of +2 sows weaned at or above their teat count. This percentage declined as initial litter size decreased, with no −1 sows that weaned at or above teat count because none in this group began with a litter size matching their teat count.

**Table 2.** Effect of initial litter size relative to functional teat count on litter performance during lactation.^a^

						*P* =		
	Initial litter size +/− functional teat count^b^					Treatment		
Item	−1	0	+1	+2	SEM	Linear	Quadratic	Parity
**Wean age, d**	21.7	22.0	22.0	21.9	0.17	0.302	0.197	0.033
**Litter size, n**								
** d 2**	13.5	14.4	15.3	16.3	0.28	<0.001	0.914	0.900
** Weaning**	12.0	12.3	12.9	13.5	0.25	<0.001	0.685	0.197
** Pigs weaned per functional teat, n**	0.831	0.858	0.901	0.945	0.0075	<0.001	0.227	<0.001
**Litter weight, kg**								
** d 2**	20.8	22.1	23.5	24.9	0.25	<0.001	0.776	<0.001
** Weaning**	78.0	78.1	80.8	83.3	1.06	<0.001	0.227	<0.001
** Change (d 2-weaning)**	57.2	56.1	57.3	58.4	1.00	0.257	0.224	<0.001
**Litter ADG, kg**	2.77	2.67	2.73	2.79	0.042	0.583	0.045	<0.001
**Mean pig BW, kg**								
** d 2**	1.54	1.53	1.53	1.53	0.016	0.417	0.850	<0.001
** Weaning**	6.51	6.35	6.27	6.17	0.063	<0.001	0.582	<0.001
**Piglet ADG, g**	230	215	211	205	2.4	<0.001	0.042	<0.001
**Litter coefficient of variation, %**								
** d 2**	14.5	14.9	15.0	14.7	1.37	0.903	0.775	0.985
** Weaning**	15.5	16.1	15.5	15.3	0.69	0.654	0.550	0.866
**On-test pig removals and mortalities, %**								
** Removals**	7.6	9.9	10.4	10.6	0.55	<0.001	0.025	0.105
** Mortality**	3.4	4.3	5.1	6.5	0.43	<0.001	0.903	0.003
** Removals and mortality**	11.1	14.2	15.7	17.2	0.65	<0.001	0.086	<0.001
** Litters with no removals or mortality**	21.8	13.0	5.6	5.2	3.17	<0.001	0.316	0.031

a A total of 1005 mixed-parity sows (Line 1050, PIC, Hendersonville TN) and litters were used from the time of cross-fostering (approximately 24-h after farrowing) until weaning. Treatment and parity category and their interaction were used as fixed effects in the statistical model. Teat count category was included as a fixed effect in the model.

b Sows were allotted to treatment at approximately 24-h after farrowing. Sows were blocked by functional teat count and parity categories and assigned to 1 of 4 treatments: one less pig than functional teat count (−1), the same number of pigs as functional teat count (0), one more pig than functional teat count (+1), or two more pigs than functional teat count (+2).

Litter weight on d 2 and at weaning increased (linear, *P <* 0.001) as initial litter size relative to functional teat count increased. While there were no differences in total litter weight gain as initial litter size relative to functional teat count increased, there was a quadratic relationship (*P = *0.045) for litter average daily gain (ADG) where pigs from −1 and +2 sows had a numerically greater increase in litter ADG compared to 0 and +1 sows. There was no difference in mean pig BW on d 2; however, pig ADG and BW at weaning decreased (linear, *P <* 0.001) as initial litter size relative to functional teat count increased.

**Fig. 3.**
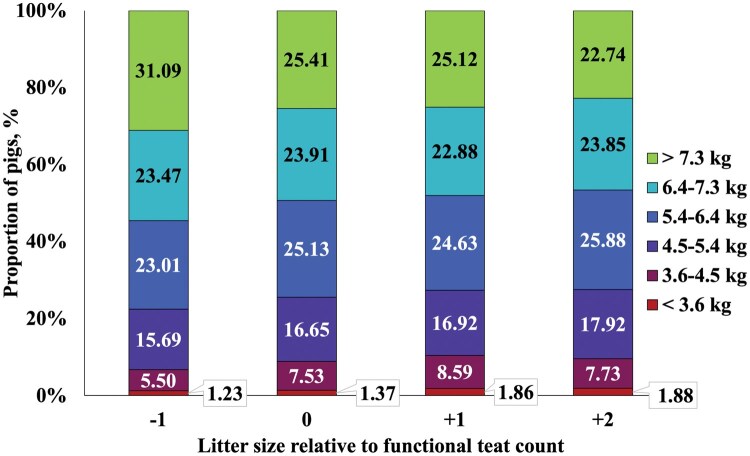
**Proportion of pigs weaned in each BW category**. Within each initial litter size relative to functional teat count treatment, the average proportion of the litter that was weaned in each BW category is shown. A total of 1005 mixed-parity sows (line 1050, PIC, hendersonville TN) and litters were used from the time of cross-fostering (approximately 24-h after farrowing) until weaning. Sows were blocked by functional teat count and parity categories and assigned to 1 of 4 treatments: one less pig than functional teat count (−1), the same number of pigs as functional teat count (0), one more pig than functional teat count (+1), or two more pigs than functional teat count (+2).

Litter weight coefficient of variation did not differ on d 2 or at weaning as initial litter size increased relative to functional teat count. When the litter was divided into the proportion of pigs in the litter weaned in pre-determined BW categories, −1 sows, had a numerically lower percentage of small pigs (<4.5 kg) at weaning and a greater proportion of pigs weaned >7.3 kg compared to other treatments (Fig. 3). However, between the other three treatments, the proportion of pigs in the litter weaned within each BW category was relatively similar. The percentage of pig removals increased (quadratic, *P <* 0.001) as initial litter size relative to functional teat count increased. The percentage of pig mortality and combined percentage of pig removals and mortalities increased (linear, *P <* 0.001) as initial litter size relative to functional teat count increased. The percentage of litters with no pig removals or mortalities decreased (linear, *P <* 0.001) as initial litter size relative to functional teat count increased.

Initial litter size relative to functional teat count affected WEI (quadratic, *P = *0.049) where +2 sows had a numerically shorter WEI compared to 0 females with −1 and +1 females intermediate (Table 3). However, the percentage of sows bred by d 7 after weaning, the subsequent farrowing rate, and the percentage of sows culled for any reason did not differ based on previous treatment. The percentage of sows culled due to failure to conceive decreased (linear, *P = *0.038) as initial litter size relative to functional teat count increased. Subsequent total born tended to increase (linear, *P = *0.061) and subsequent liveborn pigs increased (linear, *P = *0.017) as initial litter size relative to functional teat count increased. Subsequent stillbirths and mummies did not differ based on previous treatment. Estimated PSY increased (linear, *P <* 0.001) as litter size relative to functional teat count increased.

**Table 3.** Effect of initial litter size relative to functional teat count on subsequent sow performance.^a^

						*P* =		
	Initial litter size +/− functional teat count^b^					Treatment		
Item	−1	0	+1	+2	SEM	Linear	Quadratic	Parity
**Culled, %**	22.1	22.3	21.8	16.4	3.20	0.203	0.334	<0.001
** Culled due to non-conception, %**	7.7	7.9	4.1	3.3	2.17	0.038	0.728	0.047
**Wean to estrus interval, d**	5.1	5.6	5.1	5.0	0.17	0.211	0.049	0.105
**Bred by d 7 post wean, %**	86.9	83.9	89.1	90.3	2.79	0.191	0.430	0.154
**Pigs weaned/sow/year, n^c^**	29.3	30.1	31.5	32.9	0.27	<0.001	0.320	<0.001
**Farrowing rate, %**	77.7	76.1	84.7	79.1	3.38	0.319	0.431	0.179
** Total born, n**	15.9	16.5	16.5	16.8	0.37	0.061	0.584	0.002
** Liveborn, n**	14.2	15.1	15.0	15.4	0.35	0.017	0.438	0.001
** Stillborn, n**	1.1	0.9	1.0	0.9	0.16	0.372	0.626	0.592
** Mummies, n**	0.5	0.5	0.5	0.5	0.09	0.513	0.653	0.972

a A total of 1005 mixed-parity sows (Line 1050, PIC, Hendersonville TN) and litters were used from the time of cross-fostering (approximately 24-h after farrowing) until weaning. Treatment and parity category and their interaction were used as fixed effects in the statistical model. Teat count category was included as a fixed effect in the model.

b Sows were allotted to treatment at approximately 24-h after farrowing. Sows were blocked by functional teat count and parity categories and assigned to 1 of 4 treatments: one less pig than functional teat count (−1), the same number of pigs as functional teat count (0), one more pig than functional teat count (+1), or two more pigs than functional teat count (+2).

c An assumed litters/sow/year of 2.43 was utilized.

### Effect of parity category

Many of the parity category differences were typical of the differences observed among parity groups. Parity 5+ sows were heavier at entry into the farrowing house, after-farrowing, and at weaning and lost less BW during lactation and from entry to weaning (*P <* 0.001) compared to parity 1 and 2 to 4 sows (data not shown). Parity 2 to 4 sows were heavier at entry, after-farrowing, and at weaning (*P <* 0.001) compared to parity 1 sows. Parity 5+ sows had more backfat depth at entry (*P = *0.022) compared to parity 1 sows with parity 2 to 4 sows intermediate. Parity 5+ sows had more backfat depth and a greater caliper score at weaning and lost less backfat depth and fewer caliper units (*P <* 0.001) compared to parity 1 and 2 to 4 sows. Parity 2 to 4 sows had more backfat depth and greater caliper score and lost fewer caliper units over lactation (*P <* 0.001) compared to parity 1 sows. Parity 5+ sows tended to be culled at greater rates compared to parity 1 and 2 to 4 sows. Estimated PSY was greater (*P <* 0.001) for parity 1 and 2 to 4 sows than parity 5+ sows.

Parity 1 sows had decreased litter weight on d 2, litter and pig ADG, and mean pig BW on d 2 and at weaning (*P <* 0.001) compared to parity 2 to 4 and parity 5+ sows. Litter weight at weaning was greatest in parity 2 to 4 sows (*P <* 0.001) followed by parity 5+ sows and then by parity 1 sows. Parity 2 to 4 sows had increased litter weight change from d 2 to weaning (*P <* 0.001) compared to parity 1 and parity 5+ sows. The percentage of pig mortality and percentage of combined pig removals and mortality was greater in parity 5+ sows (*P ≤* 0.003) compared to parity 1 and parity 2 to 4 sows.

## Discussion

The average number of liveborn pigs per litter in the United States has risen from 11.5 in 2010 to 14.3 in 2024, with the top 10% of farms achieving 15.7 liveborn pigs per litter (PigChamp 2010, 2024). However, the average functional teat count is approximately 13.9 (Earnhardt-San et al.2023; Obermier et al.2023). The average functional teat count of sows utilized in this study averaged 14.7. Discrepancies in average functional teat count between studies may be driven by differences between genetic lines or definition of a functional teat as well as genetic progress over time. Recent estimates indicate that each additional functional teat increases litter size at weaning by an average of 0.3 pigs (Speckman et al.2021). However, the benefit varies depending on the number of pigs born alive, increasing from 0.12 pigs weaned per additional teat in litters with 10 or fewer pigs to 0.38 pigs in litters with 15 or more pigs (Speckman et al.2021).

For many years, the practice of matching the number of pigs to the number of functional teats during cross-fostering has been widely followed (Alexopoulos et al.2018). This principle was based on studies demonstrating a high level of teat fidelity observed in nursing pigs. Teat fidelity refers to the instinct of pigs to consistently nurse from the same teat throughout lactation. De Passillé et al.(1988) observed that by d 3 of lactation, teat fidelity reached 86% and by d 10, increased to 95%. It was speculated that a high level of teat fidelity was beneficial to pigs as it reduced the number of teat disputes and thus, the chance of a pig missing nursing events (De Passillé et al.1988). However, Clemons (2020) observed that pigs with intermediate nursing fidelity (51% to 80%) exhibited greater pre-weaning survival compared to their littermates with decreased or increased teat fidelity (≤50% or  ≥81%). Clemons (2020) speculated that pigs with intermediate nursing fidelity may be able to consume more milk by nursing multiple teats. The long-standing practice of matching the number of pigs to functional teats underscores the importance historically placed on minimizing competition at the udder, although in modern production systems, litter sizes often exceed the number of functional teats, prompting alternative management strategies.

When litter size exceeds the number of functional teats, producers may either employ nurse sows or allow sows to raise surplus piglets. Nurse sows typically comprise 10% to 15% of the herd, but this can increase to 43% in some circumstances (Baxter et al.2020). The use of nurse sows presents challenges, including reduced piglet growth due to lower milk yield, increased fighting, and delayed milk letdown of up to 8 h post-relocation (Kobek-Kjeldager et al.2020). Nurse sows also have greater culling rates due to reproductive failure and reduce breeding efficiency by occupying non-productive stalls (Osotsi et al.2024). Additionally, they disrupt all-in/all-out systems, increasing the risk of pathogen transmission, including PRRS and influenza A (Garrido-Mantilla et al.2020).

Previous literature that investigated the effects of litter size relative to functional teat count on PWM has yielded variable results. In litters that were equalized based on piglet BW (light or heavy), Santos et al.(2025) observed no difference in PWM in +1 sows when compared to 0 sows but observed an increase in removals and combined removals and mortality in +1 sows when compared to 0 sows. This increase in removals and combined removals and mortality is similar to results in the current study. However, the study herein used a wider range of initial litter size relative to functional teats than Santos et al.(2025) and the largest increase in removals was between the −1 and 0 treatments with a plateau between +1 and +2 sows. Similar to results of the current study, Vande Pol et al.(2021) observed an increase in PWM in +2 sows when compared to −2 sows with 0 sows intermediate.

The increase in PWM with increasing initial litter size seen in both the current study and several previous studies is likely driven by reduced access to functional teats in the first 72 h of life when the majority of PWM occurs due to starvation and crushing (Edwards and Baxter 2015; Muns et al.2016). This was observed in the present study where the majority of removals and mortalities occurred during the first week after farrowing, after which litter size remained relatively stable throughout the remainder of lactation (Fig. 1).

Regardless of whether PWM is affected, studies consistently show that litter size at weaning is increased when the number of initial pigs are increased relative to functional teat count (Vande Pol et al.2021; Willard et al.2022, 2023; Zanin et al.2024). These findings align with the current study, which also showed a linear increase in litter size at weaning as the number of pigs relative to teat count increased. Consistent with the present study, Zanin et al.(2024) and Santos et al.(2025) observed an increase in the number of pigs weaned per functional teat in +1 sows when compared to 0 sows.

While total milk yield increases with increasing litter size, milk consumption per pig declines, resulting in greater litter ADG but lower individual pig ADG (Auldist et al.1998). In the current study, litter ADG increased quadratically which indicates that the increase in piglet ADG in −1 sows was sufficient to compensate for their smaller litter size, whereas in +2 sows, the larger number of pigs contributed to greater total litter gain despite lower growth of individual piglets. Overall, these results support the expected trend that as litter size increases relative to functional teat count, litter ADG and weaning weight increase, while individual pig ADG and weaning weight decrease. The reduction in piglet ADG is likely due to lower milk intake, both from decreased milk consumption per pig and more missed nursing events by the piglet in larger litters. Santos et al.(2025) reported that +1 litters had fewer piglets that never missed a nursing event and more that missed at least one nursing event by d 5 of lactation, compared to 0 litters. Contradictory to the results herein, Santos et al.(2025) reported no difference in pig ADG, pig weaning weight, and litter weaning weight when litter size increased from being at teat count to 1 more than teat count. Similarly, when a larger range of litter size relative to functional teat count (−2, 0, or +2) was investigated, litter weaning weight and pig ADG were not different (Vande Pol et al.2021). However, Vande Pol et al.(2021) observed a tendency for reduced pig weaning weight in +2 and 0 litters compared to −2 litters.

This study assessed the proportion of pigs weaned in pre-defined BW categories based on commercial relevance. We initially hypothesized that increasing litter size relative to functional teat count may increase total number of pigs weaned but shift the BW distribution toward lighter piglets. However, the relatively small differences observed in the current study in the proportion of the litter weaned across BW categories among 0, +1, and +2 sows suggests that increasing litter size relative to teat count does not significantly increase the proportion of lightweight pigs at weaning. This interpretation is supported by the lack of difference in litter CV at weaning observed in both the current study and by Santos et al.(2025) when litter size increased relative to functional teat count. In contrast, Zanin et al.(2024) observed greater litter CV on d 5 and at weaning in +1 litters compared to 0 litters. However, the difference narrowed from 6% on d 5 to 2% at weaning, and because initial CV was not reported, interpretation remains limited.

As milk production increases with litter size (Auldist et al.1998), greater sow body condition loss is expected due to increased nutrient demands. This increase in body condition loss (BW, caliper, and backfat depth) as litter size relative to functional teat count increased was observed in the current study, though differences were modest. For example, on a percentage of BW basis, +2 sows lost only 1.4% more BW than −1 sows. Zanin et al.(2024) reported increased caliper loss but no change in backfat depth or body condition score in +1 sows compared to 0 sows. Santos et al.(2025) saw no differences in sow body condition score, caliper or backfat depth loss when +1 and 0 sows were compared, likely due to minimal differences in litter weight gain thus smaller nutrient demand differences.

Increased body condition loss during lactation can impair subsequent reproduction, particularly in parity 1 sows that are under high milk production pressure (Quesnel et al.2007). To assess differences in response to treatment based on physiology and age, parity category was included in the statistical model as an interaction with litter size relative to functional teat count and no interactions were found for measurements of subsequent reproduction. It is unclear why in the current study, sows nursing litter matching teat count actually had the numerically longest WEI and +2 sows had the shortest. Arend et al.(2023) reported no differences in WEI, percentage of sows bred by d 7, or subsequent farrowing rate in parity 1 sows when litter size was increased from 12 to 15.5. While Arend et al.(2023) did not establish litter size relative to functional teat count, on average, sows nursing 12 pigs were 2 pigs below teat count and sows nursing 15.5 pigs were at teat count. Previous research suggests BW loss must exceed 5% in primiparous and 10% in multiparous sows to impact WEI (Thaker and Bilkei 2005). In the present study, average losses across treatments were below these thresholds, likely explaining the lack of effect on subsequent reproduction measurements. While overall culling rates did not differ, fewer sows were culled due to failure to conceive in greater litter size treatments, counter to our original hypothesis that reproductive function might be affected by greater nutritional demand. Supporting this, Arend et al.(2023) found no impairment in fertility or hormonal function in primiparous sows nursing large litters, suggesting modern sows may be more resilient to lactational demands.

This study is the first to follow sows managed with varying litter sizes relative to functional teat count through a subsequent parity. Results showed a linear increase in subsequent liveborn and tendency for a linear increase in subsequent total born as initial litter size relative to teat count increased, though the underlying mechanisms remain unclear and warrant further investigation. Additionally, when calculating PSY using the herd average of 2.43 litters/sow/year, PSY also increased linearly with litter size relative to teat count, consistent with the observed increase in litter size at weaning.

In conclusion, the optimal litter size relative to functional teat count depended on the response that is of greatest interest. Sows with 1 less pig than functional teats had the lowest pig mortality and sow BW loss and greatest pig weaning weight. However, sows with 2 more pigs than functional teats had the greatest number of pigs weaned per litter, litter WW, PSY, and liveborn in the subsequent litter.

## List of abbreviations:

ADG: average daily gain
BW: body weight
CV: coefficient of variation
PSY: pigs weaned/sow/year
PWM: pre-weaning mortality
WEI: wean-to-estrus interval
WW: weaning weight
